# A Subject-Specific Kinematic Model to Predict Human Motion in Exoskeleton-Assisted Gait

**DOI:** 10.3389/fnbot.2018.00018

**Published:** 2018-04-27

**Authors:** Diego Torricelli, Camilo Cortés, Nerea Lete, Álvaro Bertelsen, Jose E. Gonzalez-Vargas, Antonio J. del-Ama, Iris Dimbwadyo, Juan C. Moreno, Julian Florez, Jose L. Pons

**Affiliations:** ^1^Cajal Institute, Spanish National Research Council (Consejo Superior de Investigaciones Científicas), Madrid, Spain; ^2^eHealth and Biomedical Applications, Vicomtech, San Sebastián, Spain; ^3^Biomechanics and Assistive Technology Unit, National Hospital for Paraplegics, Toledo, Spain; ^4^Occupational Therapy Department, Occupational Thinks Research Group, Instituto de Neurociencias y Ciencias del Movimiento, Centro Superior de Estudios Universitarios La Salle, Universidad Autónoma de Madrid, Madrid, Spain; ^5^Monterrey Institute of Technology, Monterrey, Mexico

**Keywords:** benchmarking, walking, wearable robot, rehabilitation, lower limb, skeletal modeling

## Abstract

The relative motion between human and exoskeleton is a crucial factor that has remarkable consequences on the efficiency, reliability and safety of human-robot interaction. Unfortunately, its quantitative assessment has been largely overlooked in the literature. Here, we present a methodology that allows predicting the motion of the human joints from the knowledge of the angular motion of the exoskeleton frame. Our method combines a subject-specific skeletal model with a kinematic model of a lower limb exoskeleton (H2, Technaid), imposing specific kinematic constraints between them. To calibrate the model and validate its ability to predict the relative motion in a subject-specific way, we performed experiments on seven healthy subjects during treadmill walking tasks. We demonstrate a prediction accuracy lower than 3.5° globally, and around 1.5° at the hip level, which represent an improvement up to 66% compared to the traditional approach assuming no relative motion between the user and the exoskeleton.

## Introduction

The quantitative assessment of robotic performance is a critical issue in rehabilitation robotics (Torricelli et al., 2015b). The increasing number of wearable robots available in the market has triggered the strong need for reliable methods to compare the existing solutions on a common basis. In the field of lower limb exoskeletons, devices are usually tested according to self-defined procedures and metrics that cannot be easily replicated across different laboratories and/or users. The most relevant problems are related to the intrinsic differences between devices, in terms of degrees of freedom, actuation principles, mechanisms complexity, and materials, but are also due to the heterogeneous measurement systems and protocols available worldwide. Besides this, the close interaction between the user and the robot further challenges the assessment of robotic performance independently from the user (Torricelli et al., 2015a). As a results, performance indicators normally rely on global variables such as metabolic consumption (Mooney et al., 2014; Collins et al., 2015; Galle et al., 2017), joint kinematics (Sawicki et al., 2006; Van Asseldonk et al., 2008), or spatiotemporal parameters (Buesing et al., 2015; Arazpour et al., 2016). While these approaches are effective in grasping the overall behavior of a bipedal system, they do not provide any clues on the internal mechanisms that may be relevant to the global performance, e.g., human-machine interaction (Torricelli et al., 2015a). In this respect, one aspect that has been particularly disregarded in the literature is the quantitative evaluation of human-machine kinematic compatibility. A wearable robot is, by definition, a machine that operates in constant physical contact with the human body, supporting its movement by applying forces on the subject's skin (Pons, 2008). Due to kinematic, dynamic, and morphological differences between the exoskeleton and the human body, a relative motion between them always exists. This motion is responsible for a number of disadvantages, such as energy losses during power transmission, inaccurate control of the human limbs, or discomfort and pain due to skin abrasion. During mechatronic design, the understanding of these factors is key for improving the device and its acceptance by the end user.

An accurate way to measure the relative motion between subject and exoskeleton is by means of marker-based motion capture (MOCAP) systems, which use reflective markers placed on both the exoskeleton frame and the subject limbs (Alvarez et al., 2017). This approach can produce very precise results, but requires a time-consuming experimental procedure for marker placement, post-processing, and fitting with human body models. In addition, current marker-based models are not usually compatible with the presence of an exoskeleton, leading to the need of custom-based protocols, which can be hardly replicated across different systems.

Motivated by these observations, we formulated the following question: “is it possible to predict human motion from exoskeleton motion?.” A positive answer to it would support the feasibility of estimating both exoskeleton and human motion using only the exoskeleton sensors, overcoming most of the aforementioned drawbacks. Being independent from any external measurement system, this approach would also allow measuring human-exoskeleton interaction in realistic outdoor environments.

To address this research question, we propose a modeling-experimental approach that combines personalized skeletal models of human subject with a kinematic model of the exoskeleton. We previously addressed a similar problem in the context of exoskeletons for upper limb rehabilitation (Cortés et al., 2014, 2016). In that work, we formulated and assessed a computational method, denominated EIKPE (Extended Inverse Kinematics Posture Estimation), to estimate the joint angles of the human subject when the exoskeleton motion is known. In the original version of the EIKPE, the human limb and exoskeleton are modeled as a parallel kinematic chain in which the exoskeleton's cuff constraints impose motion constraints on the human limb. Then, for a given pose of the exoskeleton, the inverse kinematics (IK) of the parallel chain was computed to find the joint angles of the subject limb during the training of single-joint (e.g., elbow flexion) or compound motions (e.g., reaching an object).

Here, we propose an extended version of the EIKPE, which adds skeletal (SK) modeling in order to improve the subject-specific prediction ability of human limb motion given the absolute pose of the exoskeleton limb. To our best knowledge, no similar approaches have been proposed in the literature.

## Materials and methods

The process of creating, applying and estimating the accuracy of the EIKPE entails the following five steps:

1. Capture of the Ground-Truth (GT) motion of the exoskeleton and human during gait. This step generates the simultaneous recording of a set of markers placed on human subjects and exoskeleton during treadmill walking.
2. Skeletal model personalization. Based on recorded GT motion of human and exoskeleton, a generic skeletal model is scaled to match the size of each test subject.
3. Human-Exoskeleton model generation. The personalized SK model of each subject is connected with a kinematic model of the exoskeleton and then the exoskeleton model link lengths are adjusted.
4. Computation of the exoskeleton and human joint angles. The GT joint angles of the human, *v*^*H*^(*t*), and exoskeleton, *v*^*R*^(*t*), are calculated using the Human-Exoskeleton model previously generated.
5. Application of the EIKPE constraints to the Human-Exoskeleton model and assessment of its accuracy in estimating human joint angles (ṽ^*H*^(*t*)) given the GT joint angles of the exoskeleton (*v*^*R*^(*t*)).

### Ground-truth motion recording

Seven healthy subjects (5 men, 2 women, age 29.7 ± 4.9) participated in the study. The experiments were performed in the Motion Analysis Laboratory of the Centro Superior de Estudios Universitarios La Salle, Universidad Autónoma de Madrid, Madrid, Spain. Subjects were asked to perform two different recording sessions. In the first session, each subject was asked to walk at 1 Km/h speed during 10 s. An additional trial was required to measure the subject in a static upright standing posture. In the second session, the subject repeated the previous trials while wearing a lower limb exoskeleton. The exoskeleton used in this experiment was the Exo-H2 (Technaid, Arganda del Rey, Spain; Bortole et al., 2015). The Exo-H2 has 6 degrees of freedom (DOFs), including hip, knee and ankle joints. Actuators are connected to each other by means of an aluminum frame with extensible plates that allow adjusting the inter-joint distance in order to adapt to a specific subject size. In this experiment, the exoskeleton was configured in “mechanically-transparent mode,” i.e., with the motors physically decoupled from the joints. In this configuration, the exoskeleton was unable to apply any assistive or resistive force at the joint level. Several belts are used to attach the exoskeleton to the subject.

**Figure 1:**
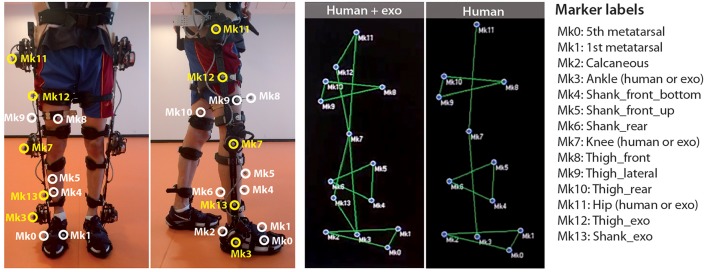
Marker placement and labeling (written informed consent was obtained from the individual for the publication of this image).

Subject and exoskeleton motion were measured by using a marker-based MOCAP system (BTS, Garbagnate Milanese, Italy) composed of eight infrared cameras. All walking trials were performed on a treadmill (LK6000 treadmill, BH fitness, Spain). A 2 cubic meter volume was previously calibrated to ensure accurate reconstruction of all markers during the experiment. Prior to the first experimental session, the subject was instrumented with 12 reflective markers of 10 mm diameter placed on different anatomical landmarks (Figure 1): three markers on the foot, placed on the calcaneus and on the fifth and first metatarsals; one marker on the malleolous; three markers on the shank; one marker on center-outside surface of the knee; three markers on the thigh; one marker on the trochanter.

After the first session, and before donning the exoskeleton, the markers on ankle, knee, and hip were removed, because the exoskeleton structure would impede their view from the cameras. The exoskeleton was equipped with five markers placed in the center-outside surfaces of the knee, ankle and hip motors, and in the midpoint of the shank and thigh bars. Motion data were recorded at 100 Hz and processed offline to obtain the labeled 3D trajectories of all markers.

### Skeletal model personalization

We generated a personalized skeletal model for each of the tested subjects by scaling a generic musculoskeletal lower limb model [model Gait2392 included in OpenSim (Yamaguchi and Zajac, 1989; Delp et al., 1990; Anderson and Pandy, 1999, 2001)], which includes 19 DOFs. Although we used a musculoskeletal model, in this work we refer to it as a skeletal model because the muscular components of the model were not used for the human kinematic estimation. The scaling and adjustment of markers were performed by using the Scale Tool of OpenSim. We have configured the Scale Tool to obtain the scaling factors along the longitudinal axis of the femur using markers Mk11 and Mk7, for the tibia using markers Mk7 and Mk3, and for the foot using markers Mk2 and Mk1 (Figure 1). For this scaling stage, we used only the static captures of the subject without the exoskeleton. Notice that with our marker protocol we were only able to scale the right lower limb. The rest of the model parts conserve the size of the original SK generic model. The results of the scaling were visually inspected. If the scaling was not coherent (e.g., the markers in the scaled model appear to be too low or high with respect to the segment they are attached to), then a manual scaling factor was applied.

### Human-exoskeleton model generation

**Figure 2:**
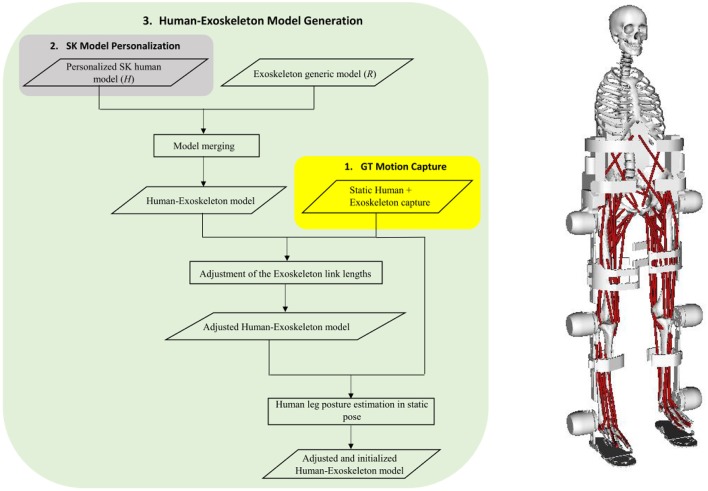
Schematic diagram of the Human-Exoskeleton model generation **(left)**, and the resulting model scaled to one real test subject **(right)**.

We modeled the fixation between the subject pelvis and the exoskeleton corset as rigid, and adjusted their relative translation such that the coordinates of the hip joint rotation centers (left and right) of the human and exoskeleton match along the anterior-posterior direction of the sagittal plane (Figure 2). Then, we adjusted the length of the exoskeleton links by using the static captures of the subjects wearing the exoskeleton, such that the axes of rotation of the joints of the hip, knee and ankle corresponded to the height indicated by markers Mk11, Mk7, and Mk3 respectively. Finally, the joint angles of the human hip (flexion, rotation, and adduction), knee (flexion) and ankle (flexion) were adjusted, using inverse kinematics, and manually revised, to match the leg posture in the static capture of the subject wearing the exoskeleton.

### Computation of the ground-truth joint angles

**Figure 3:**
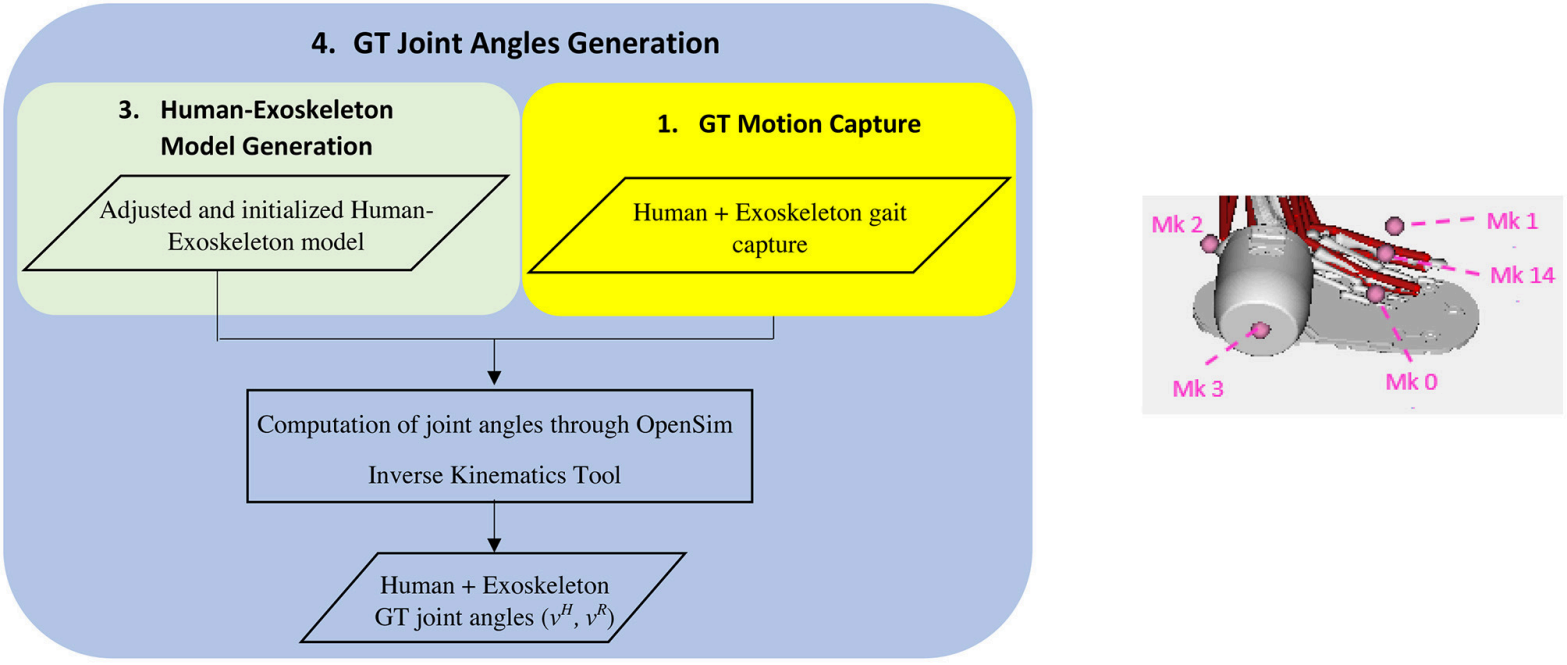
Schematic diagram of the human and exoskeleton ground-truth joint angles estimation **(left)** and detail of the markers used for the estimation of the human and exoskeleton ankle flexion **(right)**.

We computed the Ground-Truth (GT) joint angles of the human and the exoskeleton by using the Inverse Kinematics (IK) Tool of OpenSim (Figure 3). We have configured the IK tool to estimate the angles of the hip, knee and ankle for both human and exoskeleton and also the translations and rotations of the pelvis-corset junction relative to the MOCAP coordinate system. We assumed that, during the gait using the exoskeleton, the hip rotation and ab-adduction angles are like those computed at the static posture. This assumption is realistic since the exoskeleton does not include neither adduction or rotation DOFs at the hip level. To estimate the exoskeleton ankle plantar-dorsiflexion, we created a new virtual marker (Mk14, see Figure 3) located in the mid-point between markers Mk0 and Mk1. Then, the exoskeleton ankle angle can be computed using IK from markers Mk3 and Mk14.

**Figure 4:**
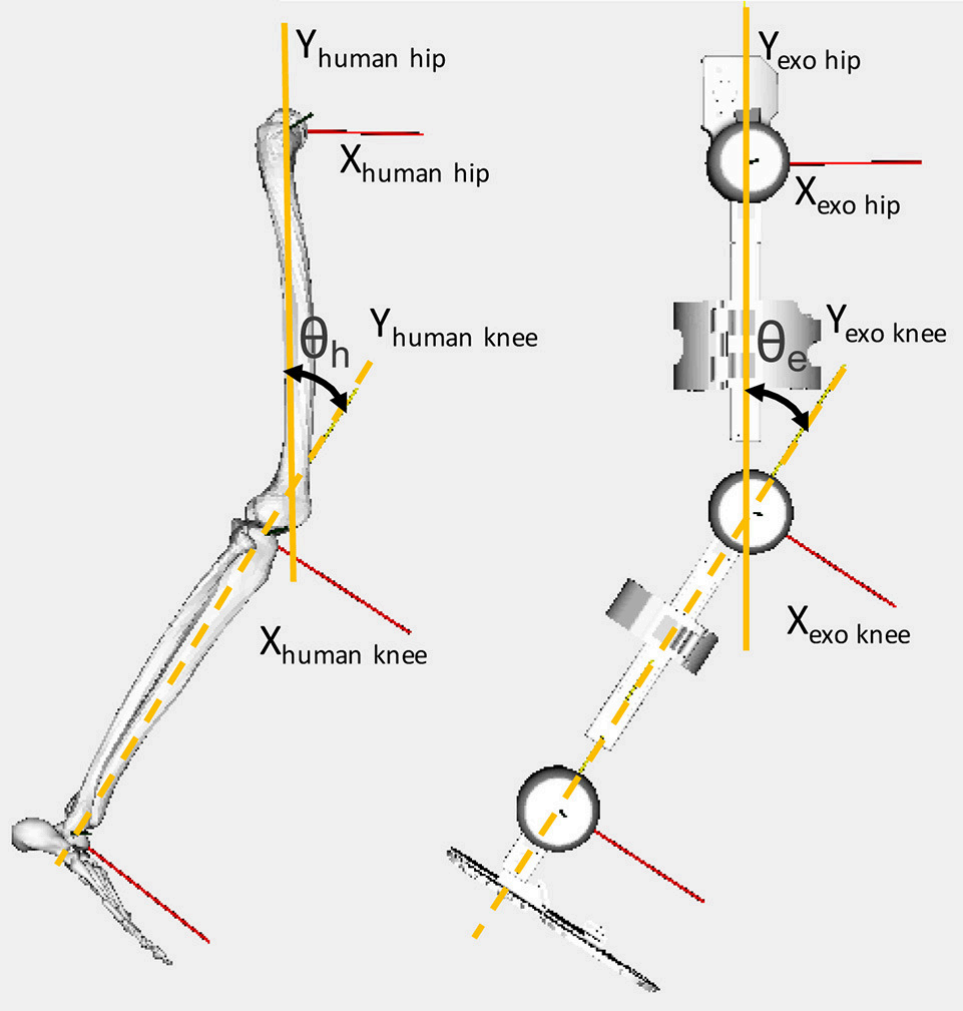
Definition of the knee flexion-extension angles for the human (θ_h_) and exoskeleton (θ_e_) models.

The GT joint angles computed correspond to the rotation around the Z axis of the joints. The neutral position of each joint is defined as the position in which the Y axis of two adjacent segments match. Figure 4 shows the definition of the knee flexion-extension for the human and exoskeleton models, as an example of the mentioned convention. The coordinate systems of the human pelvis and exoskeleton corset are aligned between them.

### Human joint angles estimation using EIKPE

**Figure 5:**
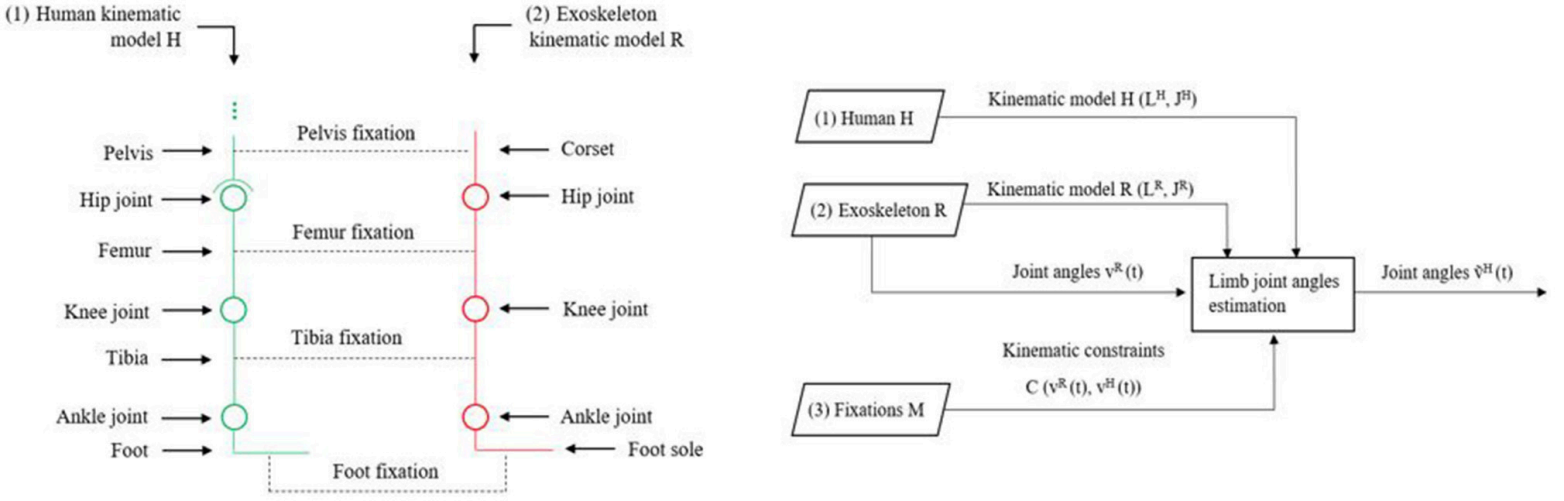
Schematic diagram of the human and exoskeleton kinematic models and their fixations **(left)** and Inputs and Outputs of the EIKPE **(right)**.

Our method to estimate the joint angles of the human lower limb during gait is based on a previous formulation of the EIKPE method (Cortés et al., 2014, 2016). In the EIKPE, the human limb and the exoskeleton are modeled as a single parallel kinematic chain connected by the fixations of the exoskeleton (Figure 5, left). For a given sequence of postures of the exoskeleton, described by its vector of joint angles *v*^*R*^(*t*), the human sequence of joint angles *v*^*H*^(*t*) is estimated.

Formally, the inputs to the problem are (Figure 5, right):

1. The human lower limb kinematic model, denoted by *H*(*L*^*H*^, *J*^*H*^) [e.g., the Denavit-Hartenberg parameters (Denavit, 1955)], where *L*^*H*^ and *J*^*H*^ correspond to sets of links and joints. The lower limb kinematic model corresponds to the personalized skeletal model obtained for each test subject.
2. The exoskeleton kinematic model, denoted by *R*(*L*^*R*^, *J*^*R*^). The values of exoskeleton joint angles *v*^*R*^ are known at any instant *t* of the gait cycle. The exoskeleton that we used in this work (H2, Bortole et al., 2015), has 6 DOFs in total, all in the sagittal plane.
3. A set of kinematic constraints, denoted *C*(*v*^*H*^(*t*), *v*^*R*^(*t*)) imposed by the human-exo fixations *M*, which are passive mechanisms that connect the exoskeleton with the human limbs. In this work, we consider the following set of constraints:
  - A 6-DOF constraint between the human pelvis and the exoskeleton corset.
  - A 2-DOF constraint between the tibia and its fixation (point-on-line constraint).
  - Three 3-DoF constraints between the human foot and the exoskeleton sole (point-to-point constraints).

The goal of the implemented algorithm is to find the approximate angles of the joints of the patient limb ṽ^*H*^(*t*), such that the set of constraints *C* are met.

**Figure 6:**
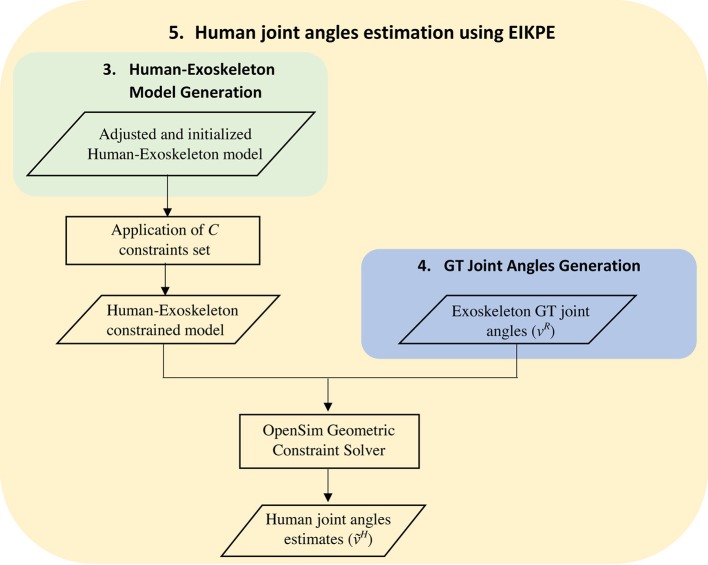
Schematic diagram of the human joint angles estimation using the EIKPE.

The application of the EIKPE to estimate *v*^*H*^(*t*) entails the following steps (Figure 6):

- Application of the set of subject-specific constraints *C* to the Human-Exoskeleton model previously determined.
- Application of the GT joint coordinates of the Exoskeleton in instant *t*_*i*_ ($v^{R}\left(t_{i}\right)$), i.e., flexion of the hip, knee, and ankle to the Human-Exoskeleton model obtained in step I.
- Estimation of the human flexion angles of the hip, knee and ankle using the geometric constraint solver of OpenSim (Delp et al., 2007) for instant $t_{i}(({\tilde{v}}^{H}\left(t_{i}\right)\;)$.
- Repetition of steps II and III for all instants *t*_*i*_ belonging to the GT dataset of the test subject.

### Assessment of the accuracy of EIKPE estimations

To assess the prediction performance, we compared the joint profiles estimated by the EIKPE with the angle estimations obtained by the GT angles from captured data. In order to determine how the EIKPE compares to the traditional rigid method, which assumes no misalignment between exoskeleton and human joint axes and segments, we compared the joint angles of the rigid method against those of the GT. The similarity of the angle estimations provided by the EIKPE and the rigid method against the GT has been assessed in terms of Root Mean Squared Error (**RMSE**) and Range of Motion Error (**ROME**) according to the following equations:

$$\begin{array}{l} RMSE\;=\sqrt{\frac{\sum_{\text{i}=1}^{n}{\left(x_{i}-y_{i}\right)}^{2}}{n}}\\ ROME=\;\left[max\left(x\right)-min\left(x\right)\right]-\;\left[max\left(y\right)-min\left(y\right)\right] \end{array}$$

where ***x*** refers to the GT human joint angles and ***y*** are the joint angles obtained by either the EIKPE or the rigid model estimator. The RMSE captures errors related to differences in the shape and offset of the estimations, whereas the ROME reflects the accuracy in the estimation of the maximum amplitude of the movement. This comparison has been performed on walking data, whereas static data have been used only for model building and calibration.

To check for statistical differences between the performance of the rigid model and the EIKPE, we conducted a Wilcoxon-Mann-Whitney test, as an alternative to the *t*-test given the low numbers of participants of this study. The test was applied on both metrics (RMSE and ROME) for each joint, and the significance was set to *p* = 0.05.

## Results

**Figure 7:**
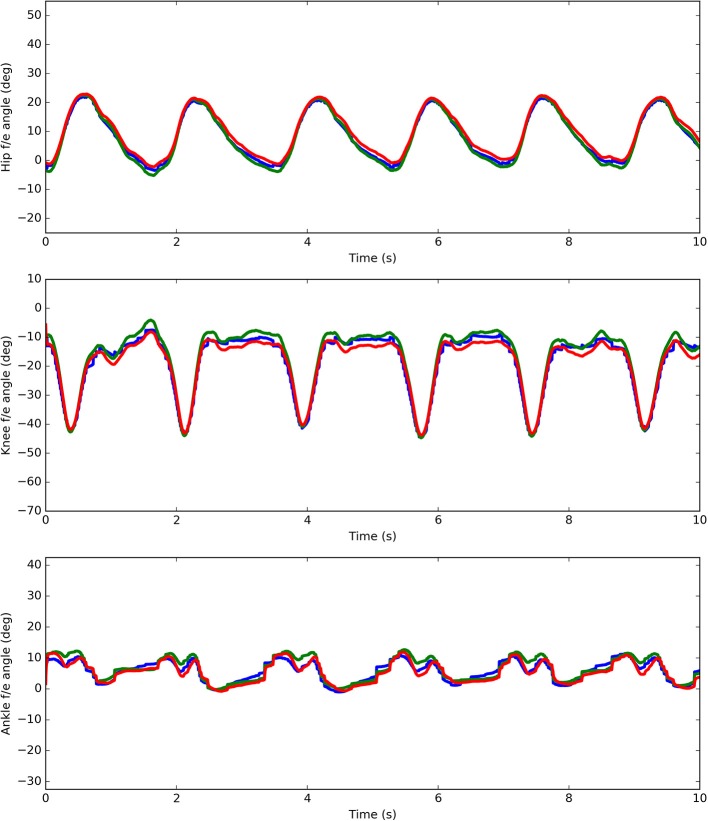
Ground-Truth (blue) and estimates (rigid model in green and the EIKPE in red) of the hip, knee, and ankle flexion-extension angles for representative gait cycles of a test subject.

Figure 7 shows the results on human joint angle estimation from one representative subject. The three profiles represent human joint angles as obtained by GT captured data (in blue), the EIKPE (in red), and the rigid model (in green). Results are given for hip, knee and ankle DOF in the sagittal plane.

**Figure 8:**
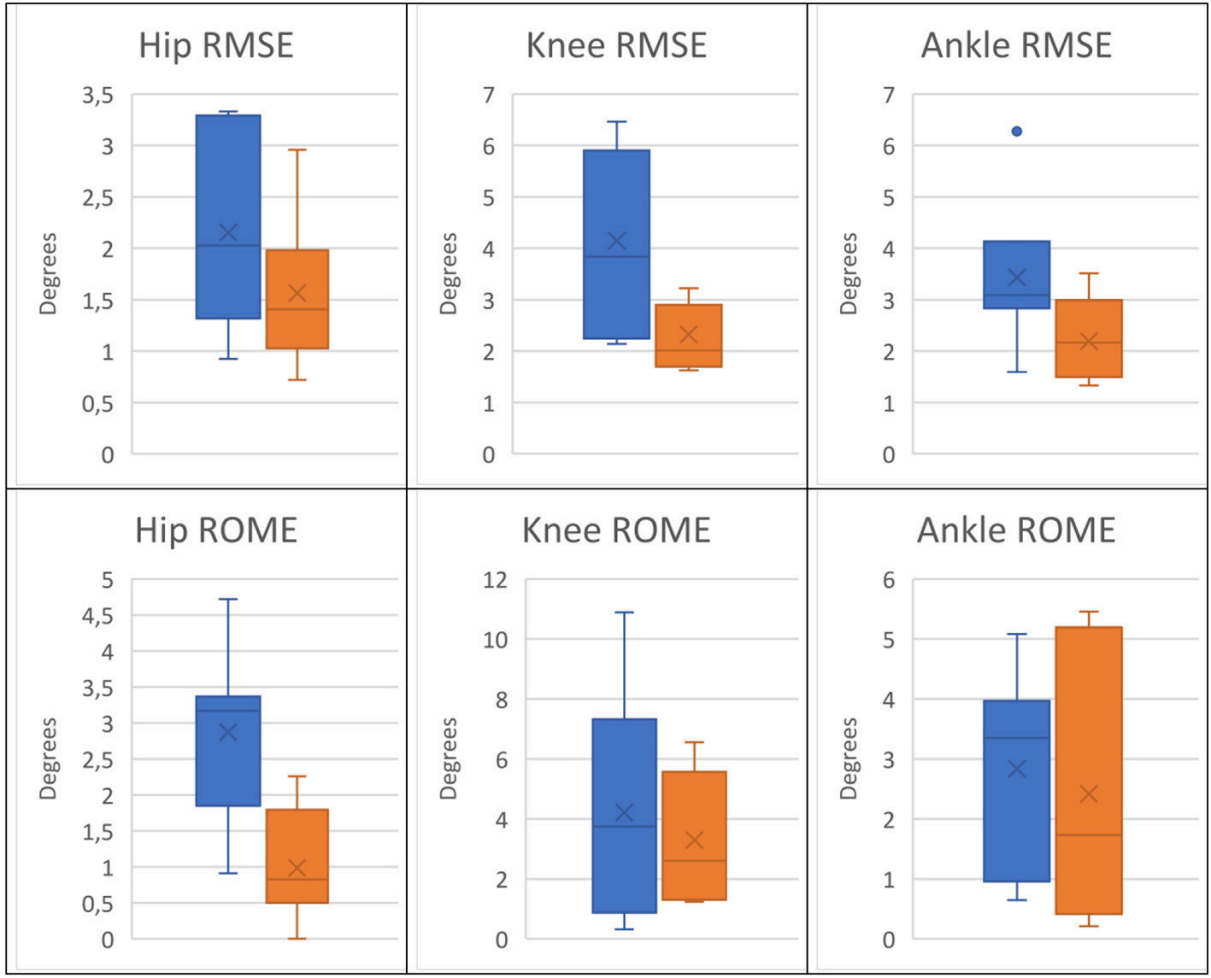
Box plots of the RMSE and ROME metrics of the angle estimations provided by the rigid model (blue) and the EIKPE (orange).

Table 1 reports the RMSE and ROME values from all the test subjects. In addition, Figure 8 presents the box-plots of the obtained values for the rigid model and the EIKPE across subjects, for each of the estimated joint angles.

**Table 1:** Human joint angle estimation errors in terms of the ROME and RMSE metrics (mean ± sd) provided by the rigid method and the EIKPE with respect to the Ground-Truth angles.

**Joint**	**Metric**	**Method**		Improvement^*^ (%)
		**Rigid Model**	**EIKPE**	
Hip	RMSE	2.2 ± 0.9	1.6 ± 0.7	27
	ROME	2.9 ± 1.2	1.0 ± 0.7	66
Knee	RMSE	4.1 ± 1.7	2.3 ± 0.7	44
	ROME	4.2 ± 3.9	3.3 ± 2.1	22
Ankle	RMSE	3.4 ± 1.5	2.2 ± 0.8	36
	ROME	2.8 ± 1.6	2.4 ± 2.1	15

* *Error reduction in the angle estimates provided by the EIKPE with respect to ones provided by the rigid model*.

When compared to the rigid model, the EIKPE showed lower errors with respect to the GT angles, in terms of both RMSE and ROME. Regarding the RMSE metric, the improvements produced by EIKPE vary between 27 and 44% while for the ROME metric vary between 15 and 66%. In particular, the hip flexion-extension showed the better estimation accuracy, with mean errors lower than 2° and a dispersion of 0.7°. For the knee and ankle, the estimation errors increased, with mean values below 3.5° for both metrics. The ankle estimations present the larger dispersion for both metrics among the studied joints (up to 2° for ROME metric). This is possibly due to cumulative errors which are amplified at the end of the human kinematic chain.

Concerning the estimations of the rigid model, the joint angle that is better estimated is the hip flexion-extension, as occurred with EIKPE, with mean errors around 2° and a dispersion near 1°. As opposed to the results obtained by the EIKPE, the knee angle presented the highest estimation errors. This result suggests that the larger misalignment between the kinematic models of the exoskeleton and human lower limb occurs at the level of the knee during gait.

Table 2 presents the *p*-values obtained from the Wilcoxon-Mann-Whitney test for RMSE and ROME. According to this analysis, in terms of RMSE, the rigid model and the EIKPE result statistically different (*p* < 0.05) only at the knee and ankle. In contrast, in terms of the ROME, statistically relevant differences are found only at the hip.

**Table 2:** Results of the Wilcoxon-Mann-Whitney test's applied to the two population of errors obtained by the EIKPE and the rigid model.

**JOINT**	**RMSE**	**ROME**
Hip	0.098	0.004^*^
Knee	0.004^*^	0.359
Ankle	0.012^*^	0.652

*The asterisk indicates a p-value lower than 0.05*.

**Figure 9:**
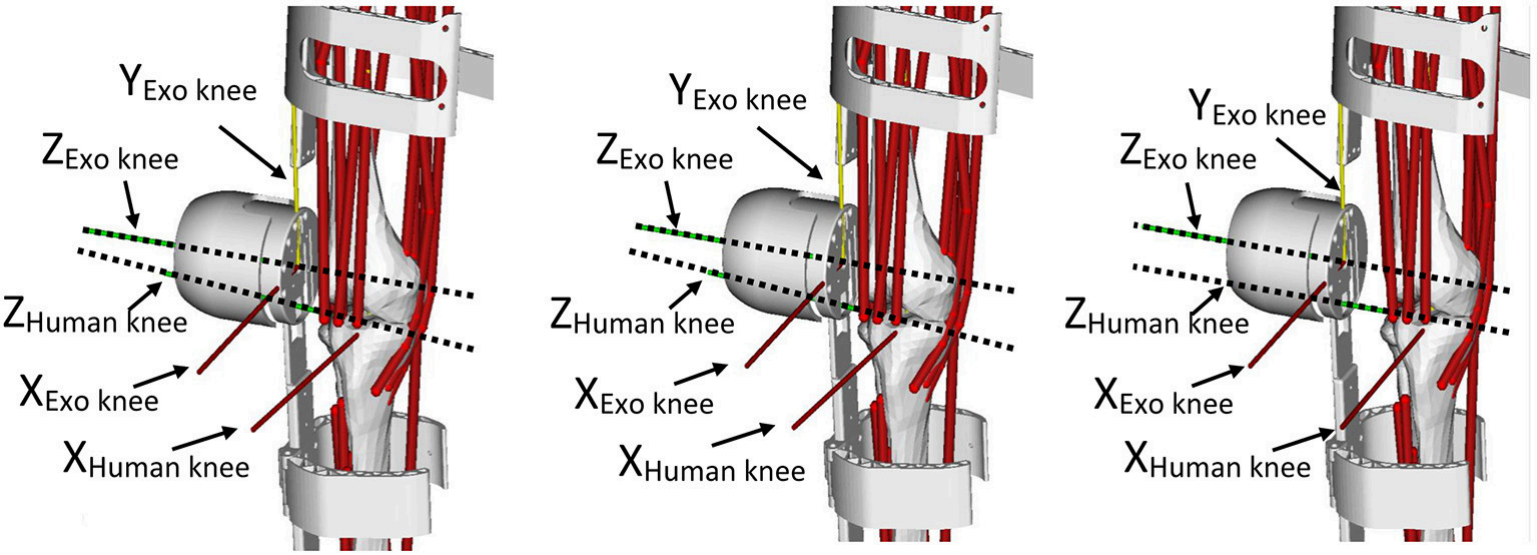
Reconstructed poses of the human lower limb at a particular phase of the gait cycle with the joint angles of the MOCAP **(left)**, the EIKPE **(middle)**, and rigid model **(right)**.

Figure 9 presents a qualitative comparison of the reconstructed poses of the human lower limb, at the knee level, during a particular phase of the gait cycle. The three figures correspond to the reconstruction using the GT joint angles (left), the EIKPE (middle), and rigid model (right) respectively. It can be observed how the rotation axes of the human (Z_Human_ _knee_) and exoskeleton knee (Z_Exo_ _knee_) differ from each other in the case of the GT data. With the rigid model, their directions are parallel, recreating an idealized and inaccurate human—exoskeleton relative pose. The EIKPE, on the contrary, is able to reconstruct the direction of Z_Human_ _knee_ close to those estimated by the GT.

## Discussion

Measuring the relative motion between human and exoskeleton is a challenging and increasingly relevant issue in wearable robotics. A recent sensitivity analysis showed that inaccurate joint angles estimates may led to inaccurate inverse dynamic estimations up to 232% during gait (Riemer et al., 2008). These sources of errors are particularly likely to happen in wearable robotics applications, where it is generally assumed that the exoskeleton and the human have negligible relative motion. This can lead to wrong estimates about power and force transmissions and therefore introduce important biases in the design of both the mechatronic components and the control paradigms of these devices. The EIKPE method here proposed showed an improvement on the accuracy of human motion prediction in the range of 15–66% over rigid model assumptions, leading to accuracies below 3° (RMSE between 1.6–2.3°). In the context of gait analysis, these figures seem to be satisfactory, being close to those obtained with other motion capture systems. For example, the method in Seel et al. (2014), based on inertial sensors, reports a RMSE between 1 and 3° for the ankle and knee flexion-extension movements, in a scenario where no exoskeletons were involved.

A major advantage of the EIKPE method is that no additional sensors apart from those embedded in the exoskeleton are required to obtain accurate estimations of the human joint angle throughout the gait cycle. This has important practical applications in clinical, industrial, and consumer domains, because they allow quick measurements in out-of-the-lab conditions. For instance, the EIKPE method can be used as a benchmark of adaptability of the exoskeleton to specific sizes of the subjects, either healthy or impaired, in the execution of real tasks. Such information can be even obtained prior to usability tests, and used to improve the design of the device, achieving better ergonomics and more efficient transmission of forces. In clinical diagnostic applications, such as during the assessment of the increment of the voluntary range of motion, the EIKPE method would enable the assessment of patient evolution during robotic intervention, and not by pre-post comparison, as currently done. The application of a method like the EIKPE becomes indispensable if the objective is the estimation of the inverse dynamics of the patient-exoskeleton system (e.g., voluntary torque applied by the patient to some joint) during the therapy. In this respect, real-time predictions of these quantities will be of invaluable help to those control strategies based on timely prediction of user intention/contribution, producing more effective assist-as-needed paradigms. Another advantage of the proposed method is to rely on purely geometrical algorithms. This means that the constraints between relative positions/orientations of exoskeleton and subject limbs are valid independently from the correct execution of walking movements. Therefore, the method is expected to be robust to different motor tasks or to the execution of abnormal movement patterns such as those experience in clinical cases. However, these aspects are still to be confirmed experimentally.

The model here proposed presents a number of simplifications that need to be considered when assessing the generalization of the results.

First, the personalization of our skeletal model is currently based on scaling factors obtained from the lengths of femur, tibia and foot of the subject. Therefore, the model is not able to account to subject-specific deformities (e.g., those experienced by cerebral palsy patients). In addition, this personalization process is not fully automatic. The intervention of a human operator is required to check and adjust, if necessary, the results of the inverse kinematics (IK), after joining the human skeletal model with the exoskeleton. If this initialization is poor, the EIKPE will produce estimations that do not match the shape of the angle vs. time curves of the GT, introducing a constant bias. This issue is due to the unavoidable absence of markers on the human hip, knee, and ankle joints when the subject is wearing the exoskeleton.

The second limitation is related to the kinematic constraints between the human and the exoskeleton. In the current implementation, these constraints are constant over time and do not account for the compliant behavior of soft biological tissues or other non-rigid exoskeletons components, such as braces. These elements change their shapes under the effect of interaction forces, e.g., during changes in walking speed or level of robot assistance. In these cases, the relative movement between the exoskeleton and the human may diverge from the one estimated by the EIKPE.

The third limiting factor is represented by the limited sample size of the experiment performed, which included seven healthy people, and the motion considered, limited to treadmill walking. Larger experimental studies with higher number of people, including patients, and on different tasks are required to validate the suitability of our methodology for industrial (e.g., human capability enhancement) and clinical (e.g., neurorehabilitation) applications.

The aforementioned considerations, while showing the limits of our approach, also provide clear indications that, even in presence of strong simplifications, modeling the relative motion between the human and the robot produce significantly better results than conventional “rigid” approaches. This, in our opinion, represents important evidence that supports and motivates the following next research steps in this complex and emerging field: (a) the study the sensitivity of EIKPE to non-modeled dynamic behaviors, e.g., the elasticity of exoskeleton braces or soft tissues, (b) the generation of personalized musculoskeletal models from medical images (e.g., computerized tomography) to improve the prediction accuracy in presence of bone deformities present in specific populations, e.g., cerebral palsy, (c) the implementation of a real-time version of the EIKPE with musculoskeletal models that can be used in the control loop of the exoskeleton, (d) the inclusion and testing of new predictive models of interaction forces, including models of soft tissues and robotic compliant elements, (e) testing the accuracy of the EIKPE across different type of motor tasks, e.g., slopes, sit-to-stand, rough terrains.

## Conclusions

In this work, we presented a methodology, called EIKPE, that allows to generate subject-specific skeletal models to quantify the human-exoskeleton interaction at kinematic level. We have implemented a version of the EIKPE for the lower limb with the objective of testing whether such model allows to predict, with sufficient precision, the human joint motion starting from the knowledge of the exoskeleton motion. We have assessed, in terms of the RMSE and ROME metrics, the estimation errors of the EIKPE with respect to real motion of seven healthy subjects, and compared them with a traditional rigid model that assumed no relative motion between human and exoskeleton. Our results suggest that EIKPE can be used to predict human motion from exoskeleton motion, providing estimates close to the real joint angles calculated from motion capture data. Compared to the rigid model, the EIKPE demonstrated improvements in range of 15–66% in the RMSE and ROME, depending on the joint considered. This method has several potential applications in real scenarios, e.g., assessing of the adaptability of a particular exoskeleton to specific subjects, monitoring the human-machine interaction in real-time, or improving assist-as-needed control strategies.

## Ethics statement

This study was carried out in accordance with the recommendations of Research Committee of the National Hospital for Paraplegics. The protocol was approved by the Ethics Review Board of Complejo Hospitalario de Toledo. All subjects gave written informed consent in accordance with the Declaration of Helsinki.

## Author contributions

DT and CC: contributed equally to the development and writing of this work; ÁB, JG-V, Ad-A, JM, JF, and JP: contributed to the conception of the presented work, theory verification, and supervision of the project; DT and ID: contributed to the experimental setup, subject recording, and data review; CC, NL, and ÁB: contributed to the implementation of the methods, data processing, and article writing. All authors discussed the results and contributed to the final manuscript.

### Conflict of interest statement

The authors declare that the research was conducted in the absence of any commercial or financial relationships that could be construed as a potential conflict of interest.

## References

[B1] Alvarez M. T. Torricelli D. del-Ama A. J. Pinto D. Gonzalez-Vargas J. Moreno J. C. (2017). Simultaneous estimation of human and exoskeleton motion: a simplified protocol, in 2017 International Conference on Rehabilitation Robotics (ICORR) (London, UK: IEEE), 1431–1436. (Accessed December 10, 2017). 10.1109/ICORR.2017.8009449 28814021

[B2] Anderson F. C. Pandy M. G. (1999). A dynamic optimization solution for vertical jumping in three dimensions. Comput. Methods Biomech. Biomed. Engin. 2, 201–231. 10.1080/10255849908907988 11264828

[B3] Anderson F. C. Pandy M. G. (2001). Dynamic optimization of human walking. J. Biomech. Eng. 123, 381–390. 10.1115/1.1392310 11601721

[B4] Arazpour M. Moradi A. Samadian M. Bahramizadeh M. Joghtaei M. Ahmadi Bani M.. (2016). The influence of a powered knee–ankle–foot orthosis on walking in poliomyelitis subjects: a pilot study. Prosthet. Orthot. Int. 40, 377–383. 10.1177/0309364615592703 26184037

[B5] Bortole M. Venkatakrishnan A. Zhu F. Moreno J. C. Francisco G. E. Pons J. L.. (2015). The H2 robotic exoskeleton for gait rehabilitation after stroke: early findings from a clinical study. J. Neuroeng. Rehabil. Biomed Central 12, 54. 10.1186/s12984-015-0048-y 26076696 PMC4469252

[B6] Buesing C. Fisch G. O'Donnell M. Shahidi I. Thomas L. Mummidisetty C. K.. (2015). Effects of a wearable exoskeleton stride management assist system (SMA®) on spatiotemporal gait characteristics in individuals after stroke: a randomized controlled trial. J. Neuroeng. Rehabil. 12, 69. 10.1186/s12984-015-0062-0 26289955 PMC4545867

[B7] Collins S. H. Wiggin M. B. Sawicki G. S. (2015). Reducing the energy cost of human walking using an unpowered exoskeleton. Nature 522, 212–215. 10.1038/nature14288 25830889 PMC4481882

[B8] Cortés C. Ardanza A. Molina-Rueda F. Cuesta-Gómez A. Unzueta L. Epelde G.. (2014). Upper limb posture estimation in robotic and virtual reality-based rehabilitation. Biomed. Res. Int. 2014:82190. 10.1155/2014/821908 25110698 PMC4119692

[B9] Cortés C. de los Reyes-Guzmán A. Scorza D. Bertelsen Á. Carrasco E. Gil-Agudo Á.. (2016). Inverse kinematics for upper limb compound movement estimation in exoskeleton-assisted rehabilitation. Biomed. Res. Int. 2016, 1–14. 10.1155/2016/2581924 27403420 PMC4925945

[B10] Delp S. L. Anderson F. C. Arnold A. S. Loan P. Habib A. John C. T.. (2007). OpenSim: open-source software to create and analyze dynamic simulations of movement. IEEE Trans. Biomed. Eng. 54, 1940–1950. 10.1109/TBME.2007.901024 18018689

[B11] Delp S. L. Loan J. P. Hoy M. G. Zajac F. E. Topp E. L. Rosen J. M. (1990). An interactive graphics-based model of the lower extremity to study orthopaedic surgical procedures. IEEE Trans. Biomed. Eng. 37, 757–767. 10.1109/10.102791 2210784

[B12] Denavit J. (1955). A kinematic notation for lower-pair mechanisms based on matrices. Trans. ASME. J. Appl. Mech. 22, 215–221.

[B13] Galle S. Malcolm P. Collins S. H. De Clercq D. (2017). Reducing the metabolic cost of walking with an ankle exoskeleton : interaction between actuation timing and power. J. Neuroeng. Rehabil. 14, 35. 10.1186/s12984-017-0235-0 28449684 PMC5408443

[B14] Mooney L. M. Rouse E. J. Herr H. M. (2014). Autonomous exoskeleton reduces metabolic cost of human walking during load carriage. J. Neuroeng. Rehabil. 11:80. 10.1186/1743-0003-11-80 24885527 PMC4036406

[B15] Pons J. L. (2008). Wearable Robots: Biomechatronic Exoskeletons. Chichester, UK: Wiley. (Accessed December 7, 2010).

[B16] Riemer R. Hsiao-Wecksler E. T. Zhang X. (2008). Uncertainties in inverse dynamics solutions: a comprehensive analysis and an application to gait. Gait Posture 27, 578–588. 10.1016/j.gaitpost.2007.07.012 17889542

[B17] Sawicki G. S. Domingo A. Ferris D. P. (2006). The effects of powered ankle-foot orthoses on joint kinematics and muscle activation during walking in individuals with incomplete spinal cord injury. J. Neuroeng. Rehabil. 3:3. 10.1186/1743-0003-3-3 16504172 PMC1435761

[B18] Seel T. Raisch J. Schauer T. (2014). IMU-based joint angle measurement for gait analysis. Sensors 14, 6891–6909. 10.3390/s140406891 24743160 PMC4029684

[B19] Torricelli D. Del Ama A. J. Gonzalez J. Moreno J. Gil A. Pons J. L. (2015a). Benchmarking lower limb wearable robots: Emerging approaches and technologies, in 8th ACM International Conference on PErvasive Technologies (Corfu).

[B20] Torricelli D. Gonzalez-Vargas J. Veneman J. F. Mombaur K. Tsagarakis N. Moreno J. C. (2015b). Benchmarking bipedal locomotion. A unified scheme for humanoids, wearable robots, and humans. IEEE Robot. Autom. Mag. 22, 103–115. 10.1109/MRA.2015.2448278

[B21] Van Asseldonk E. H. F. Veneman J. F. Ekkelenkamp R. Buurke J. H. Van Der Helm F. C. T. Van Der Kooij H. (2008). The effects on kinematics and muscle activity of walking in a robotic gait trainer during zero-force control. IEEE Trans. Neural Syst. Rehabil. Eng. 16, 360–370. 10.1109/TNSRE.2008.925074 18713676

[B22] Yamaguchi G. T. Zajac F. E. (1989). A planar model of the knee joint to characterize the knee extensor mechanism. J. Biomech. 22, 1–10. 10.1016/0021-9290(89)90179-6 2914967

